# The Prevalence of Malaria Among Pregnant Women at a Tertiary Care Hospital in Somalia: A Cross-Sectional Study

**DOI:** 10.1155/bmri/6730167

**Published:** 2025-08-24

**Authors:** Said Mohamed Mohamud, Serpil Doğan, Ahmed Issak Hussein, Rahma Yusuf Haji Mohamud, Zerife Orhan, Adem Doğaner

**Affiliations:** ^1^Department of Medical Microbiology, Mogadishu Somalia Turkiye Recep Tayyip Erdogan Training and Research Hospital, Medical Microbiology Laboratory, Mogadishu, Somalia; ^2^Department of Obstetrics and Gynecology, Mogadishu Somalia Turkiye Recep Tayyip Erdogan Training and Research Hospital, Medical Microbiology Laboratory, Mogadishu, Somalia; ^3^Department of Nursing, Mogadishu Somalia Turkiye Recep Tayyip Erdogan Training and Research Hospital, Mogadishu, Somalia; ^4^Department of Medical Services and Techniques, Vocational School of Health Services, Kahramanmaraş Sütçü Imam University, Kahramanmaraş, Türkiye; ^5^Department of Biostatistics and Medical Informatics, Faculty of Medicine, Kahramanmaraş Sütçü Imam University, Kahramanmaraş, Türkiye

**Keywords:** malaria, pregnant women, prevalence, Somalia

## Abstract

**Background:** In countries like Somalia, where health infrastructure is inadequate and malaria is endemic, immunosuppression during pregnancy increases the risk of placental malaria; this, in turn, leads to anemia, low birth weight, preterm delivery, and stillbirth, causing severe complications that pose a life-threatening risk to both the mother and fetus.

**Aim:** The aim of this study was to investigate the prevalence and associated factors of malaria parasitemia among pregnant women attending the obstetric clinic of a tertiary hospital in Somalia.

**Method:** This cross-sectional study, conducted from November 2022 to January 2023 at a tertiary hospital in Mogadishu, involved 398 pregnant women. Both symptomatic and asymptomatic participants were screened for malaria using Giemsa-stained blood smear microscopy. Associations between variables and malaria prevalence were analyzed using IBM SPSS 22 with chi-square tests and logistic regression.

**Results:** Among 398 pregnant women, 238 (59.8%) tested positive for malaria. Of the 238 malaria cases, 218 (91.6%) were revealed as *Plasmodium falciparum* cases. Being in the second trimester (OR: 0.524, 95% CI: 0.279–0.983; *p* = 0.044), third trimester (OR: 0.442, 95% CI: 0.245–0.797; *p* = 0.007), and indoor residual spraying (IRS) (OR: 0.192, 95% CI: 0.108–0.342; *p* < 0.001) were significantly associated with decreased odds of malaria in pregnancy.

**Conclusion:** The study revealed a high prevalence of malaria in pregnancy, predominantly caused by *Plasmodium falciparum*. Targeted education campaigns focusing on women in the second trimester and those not using preventive measures such as IRS or ITNs should be prioritized, along with routine malaria screening at every antenatal care visit.

## 1. Introduction

Malaria is an infection transmitted to humans through the bite of *Anopheles* mosquitoes, accompanied by seizures, fever, anemia, and splenomegaly [1]. Malaria is caused by intracellular parasites belonging to five species of the Plasmodium genus: *Plasmodium falciparum* (*P. falciparum*), *Plasmodium vivax* (*P. vivax*), *Plasmodium ovale*, *Plasmodium malariae*, and *Plasmodium knowlesi* (*P. knowlesi*) [2] Blood is the food that *Anopheles* mosquitoes need to survive and lay eggs. The infectious sporozoites from the salivary gland of the infected female *Anopheles* mosquito enter human circulation during feeding [3]. Once injected, parasites move through the bloodstream and go through one cycle of development if they make it to the liver. After this, the parasites enter the red blood cells and begin to grow there, causing recognizable symptoms [4].

Despite global progress in malaria control, sub-Saharan Africa still bears a disproportionate burden, accounting for 93% of global malaria cases and 94% of deaths [5].

An estimated 10,000 maternal and 100,000 newborn deaths globally were attributed to malaria, with more than half of these cases occurring in sub-Saharan Africa [6].

Malaria remains one of the most serious public health problems in many low- and middle-income countries [7]. Pregnant women and children under 5 years of age are at higher risk of contracting the disease, and the parasites are aggressive in these population groups [8, 9]. It has been documented that pregnant women are more susceptible to malaria because of their altered immune responses and physiological changes [10]. Factors outside the health sector, including social determinants such as economic status, are also important drivers of malaria in these settings [7]. Malaria during pregnancy can cause many maternal and fetal complications, including miscarriage, stillbirth, intrauterine growth restriction (IUGR), preterm birth, low birth weight (LBW), and maternal death [11].

Malaria is diagnosed by microscopic examination, molecular methods, and rapid diagnostic assays. The most common diagnostic technique for finding *Plasmodium* parasites in healthcare institutions across the globe is light microscopy, which finds parasites in capillary or venous blood [12]. Early diagnosis and treatment act an important role in malaria prevention and control. The first-line ant-malaria treatments for *P. falciparum* and *P. vivax*, respectively, are combination therapy based on artemisinin and chloroquine [13]. The World Health Organization has developed and recommended intermittent preventive therapy of malaria in pregnancy using sulfadoxine–pyrimethamine, especially in malaria-endemic countries, to lessen the health impact of malaria on pregnant women, their fetus, and newborns [14].

This global and pregnancy-focused challenge is especially acute in Somalia, where systemic and infrastructural issues, such as years of civil war, political instability, and poor health infrastructure, have hampered effective malaria control. Given these, malaria remains endemic, particularly in Mogadishu, where urban crowding and poor sanitation contribute to persistent transmission [15].

In Somalia, where malaria transmission is seasonal and often underreported, pregnant women remain a high-risk group, but local data on prevalence are lacking due to the lack of evidence-based studies focusing on rural or urban disparities. Given the vulnerability of pregnant women to adverse outcomes, this study is aimed at determining the prevalence and associated factors of malaria parasitemia among pregnant women attending a tertiary hospital in Mogadishu, Somalia.

Although malaria is endemic in Somalia, few epidemiological studies have examined its burden specifically among pregnant women. This data gap limits the development of evidence-based policies for maternal health in the region. Findings from this study will contribute to the WHO Global Technical Strategy for Malaria (2016–2030), which emphasizes the importance of targeting high-risk populations, including pregnant women in endemic areas. In alignment with these global goals, the findings may also inform national health policies by providing locally relevant evidence to guide targeted malaria prevention efforts among pregnant women in Somalia.

## 2. Materials and Methods

### 2.1. Study Setting and Population

The study was conducted in Mogadishu, the capital city of Somalia, located in the southeastern region of the country. The city experiences a hot, arid climate with limited rainfall and an average annual temperature of approximately 30°C, conditions conducive to malaria transmission.

### 2.2. Population and Sample

This cross-sectional study was conducted in the Medical Microbiology and Obstetrics Departments of Mogadishu Somali Türkiye Training and Research Hospital from November 2022 to January 2023. In this study, 398 randomly selected symptomatic or nonsymptomatic pregnant women who were admitted to the Gynecology Clinic of our hospital and who agreed to participate in the study were included.

### 2.3. Inclusion and Exclusion Criteria and Informed Consent

Pregnant women attending the gynecology outpatient clinic during the study period were eligible if they provided informed consent, regardless of symptom status or gestational age. Women with serious unrelated comorbidities (e.g., HIV and tuberculosis), incomplete clinical records, or who declined participation were excluded. Informed consent was obtained in written form. For participants with limited literacy, trained staff explained the study verbally in the local language, and verbal confirmation was followed by signature or thumbprint, in line with ethical research standards.

### 2.4. Data Collection

The study collected data on sociodemographic characteristics and other factors associated with malaria symptoms. For this purpose, a structured 8-variable interviewer-administered questionnaire, obtained by the researchers through a literature review, was used. Before the data were collected, a preliminary test was conducted on 10 patients by the researchers; the data were started to be collected after the necessary corrections on the questionnaire. These variables include gravidity, trimester, age, marital status, residence, education level, regular use of insecticide-treated bed net (ITN) utilization, symptomatic or asymptomatic. The patients' hemoglobin values and blood groups were obtained from the electronic file records in the hospital information system. Written consent was obtained from the participants for our study, and they were asked to answer the survey on a voluntary basis. Our study was conducted in accordance with the Declaration of Helsinki.

### 2.5. Sample Collection and Method

First, the third finger of the left hand of symptomatic and asymptomatic pregnant women who applied to our hospital's obstetrics and gynecology clinic was wiped with a piece of cotton lightly soaked in alcohol. Capillary blood was taken by finger prick, and thick drop and thin smear preparations were prepared from these blood samples. Slides were stained with the Giemsa method and examined under oil immersion (100× objective) by two independent laboratory technicians. Discordant readings were resolved by a third reader. It was evaluated for the presence of trophozoites, schizonts, and gametocytes belonging to *Plasmodium* species. While at least 200 microscopic areas were scanned in thin smear blood preparations, the diagnosis was made by scanning at least 100 microscopic areas in thick blood drop preparations [16].

### 2.6. Statistical Analysis

Data were entered with SPSS Version 22 (IBM SPSS for Windows Version 22, IBM Corporation, Armonk, New York, United States) software and analyzed using both descriptive and inferential statistics. Statistical parameters of qualitative variables are expressed as ratios (%) and frequencies (*n*). Associations were assessed using chi-square or Fisher's exact tests. Binary logistic regression was used to identify predictors of malaria parasitemia, with odds ratios (ORs) and 95% confidence intervals (CIs) reported. A *p* value < 0.05 was considered statistically significant.

## 3. Results

### 3.1. Sociodemographic Profile

Table 1 shows the sociodemographic and disease-related characteristics of the participants. Among 398 pregnant women, 238 (59.8%) tested positive for malaria. Of these, 218 (91.6%) were *P. falciparum* cases and 20 (8.4%) were *P. vivax* cases. Then, 242 (60.8%) of the pregnant women are in the 21–30 age group. This suggests that the risk of malaria infection primarily affects women at the peak of their reproductive age. A large proportion (67.6%) were multigravida, reflecting a population with repeated pregnancy exposure and possibly cumulative malaria risk. Then, 187 (47.0%) of the participants were in the second trimester and 250 (62.8%) of them had a blood type of 0 Rh+. While almost all of the pregnant women, 382 (96.0%), reside in urban areas, the rates of those with no education and those who are college graduates are close to each other (31.7% and 31.9%, respectively). Additionally, 76.7% of participants reported a history of malaria, suggesting recurrent exposure. Notably, 67.1% did not use any malaria prevention methods, indicating a gap in protective behavior. The most commonly reported symptoms were headache (47.7%) and fever (30.4%). Anemia was present in 65.6% of the women, highlighting a common comorbidity in this population.

### 3.2. Prevalence of Malaria, Trimester, Prevention Methods, Symptoms and Relationship With Other Factors

Table 2 shows the factors affecting malaria in pregnant women. Malaria positivity was most common among women aged 21–30 years (63.0%) and least common in the 41–50 age group (1.3%). Multigravidas had a higher positivity rate (65.1%) than primigravidas (34.9%). The highest prevalence was observed in the second trimester (51.3%), and the lowest in the third trimester (15.5%). Pregnant women with blood group O Rh+ showed a higher infection rate (66.4%) than other groups. Malaria prevalence was similar among women with secondary education (31.5%) and those without formal education (31.1%), while the lowest rate was seen in university graduates (10.5%). Among malaria-positive women, 75.2% had a prior history of malaria, and 71.2% were not using any preventive methods. Infection was significantly more common in symptomatic women (71.8%) compared to asymptomatic ones (28.2%), and 67.6% of infected women were anemic. Statistically significant associations were found for trimester (*p* = 0.011), use of preventive measures (*p* < 0.001), and symptom status (*p* < 0.001). No significant associations were observed with age, gravidity, blood group, marital status, residence, education, previous malaria, or anemia status.

### 3.3. Multivariate Logistic Regression Analysis of Malaria Risk Factors

Logistic regression analysis is given in Table 3. According to this analysis in Table 3, being in the second trimester was significantly associated with lower odds of malaria infection compared to the first trimester (OR: 0.524, 95% CI: 0.279–0.983; *p* = 0.044). Similarly, women in the third trimester had even lower odds of malaria infection compared to those in the first trimester (OR: 0.442, 95% CI: 0.245–0.797; *p* = 0.007). Regarding preventive measures, pregnant women who used indoor residual spray (IRS) had significantly reduced odds of malaria infection compared to those who used no preventive method (OR: 0.192, 95% CI: 0.108–0.342; *p* < 0.001). In contrast, the use of ITNs was not statistically significant (OR: 0.714, 95% CI: 0.340–1.502; *p* = 0.375). Other variables such as age group, gravidity, blood group, residence, and anemia status did not show statistically significant associations with malaria infection (*p* > 0.05 for all).

## 4. Discussion

In underdeveloped countries, malaria continues to be a major cause of morbidity and mortality, especially for children and pregnant women [17]. In this study, the overall prevalence of malaria during pregnancy was determined as 59.8% (Table 1). This result was similar to the prevalence of malaria in pregnant women (59.9%) found in a study in Eastern Nigeria [18] suggesting a shared epidemiological profile possibly influenced by comparable climate and healthcare access challenges. But it is lower than prevalences found in studies in Abia State, Nigeria (68.4%) [19] and Southwestern Nigeria (72%) [20]. However, positivity rates were higher than studies conducted in Southern Laos (8.3%) [21], Western Ethiopia (10.2%) [22], Burkina Faso (18.1%) [23], Northwestern Uganda (26.1%) [24], and southwestern Nigeria (40.2%) [25]. This difference in prevalence rates in the studies can be explained by the different geographical locations of the study areas. For example, our study was conducted in a region where malaria transmission is high. In addition, the study has been conducted with both symptomatic and asymptomatic pregnant women, which may have resulted in a high prevalence of malaria in pregnant women. In studies that included only asymptomatic pregnant women [26], the prevalence was found to be quite low (2.74%). Another reason for this difference may be the differences in diagnostic approaches [27] and the seasonal differences such as rainfall and temperature in the regions where the studies were conducted. In a study conducted in Ethiopia during the months of February–April (dry season), when malaria transmission is low, malaria prevalence was found to be relatively low (20.8%) [28]. However, the fact that this study was conducted during the seasons (November–January) when malaria transmission increases and that the majority of pregnant women participating in the study did not use any method (IRS or ITN) to prevent malaria may have been effective in our high prevalence.

In this study, *P. falciparum* cases were found to be 91.6%. In studies conducted in various sub-Saharan African countries, *P. falciparum* was reported as the dominant species, with rates of 42.6% in Southern Nigeria [29], 65.0% in Northeastern Ethiopia [30], and 12.2% in Ethiopia [28].

In our study, while the incidence of malaria parasite during pregnancy was seen to be higher in the 21–30 age group (63.0%) and lower in the 41–50 age group (1.3%) compared to other age groups, no significant relationship was found between maternal age and the prevalence of malaria during pregnancy. In another study [24], no significant relationship was found between maternal age and the prevalence of malaria during pregnancy, similar to our results, while some studies reported a relationship between young maternal age (< 20 years) and malaria during pregnancy [31, 32]. The relationship between young maternal age and malaria in pregnancy is thought to be related to the level of acquired malaria immunity, which, according to the literature, increases with the frequency of infection in consecutive pregnancies. It might also imply that younger women are more likely than older ones to become first-time moms; hence, they might be more vulnerable to malaria throughout their pregnancies than older women who might have experienced many infections [24].

Another important factor examined in this study was the relationship between gravidity and malaria prevalence. A meta-analysis in sub-Saharan Africa found that primigravidae mothers were more likely to acquire *P. falciparum* infection than multigravidae mothers. This suggests that variant-specific immune responses develop following exposure during pregnancy in multigravidae mothers [28]. Similar associations were found in studies done in sub-Saharan Africa [33], Ghana [34], and Burkina Faso [35]. Unlike these studies, our study found that multigravidas (65.1%) had a higher malaria prevalence than primigravidas (34.9%). However, this increase was not statistically significant (Table 2). Although contrary to the consensus that primigravidas are at higher risk due to lack of acquired immunity, our finding of higher prevalence in multigravidas may reflect increased cumulative exposure or sociobehavioral factors such as care-seeking delays in later pregnancies. Additionally, the reason for this difference can be explained by the high birth rates and the high number of multigravida pregnancies in the region where the study was conducted.

The variation in malaria prevalence across the trimesters of pregnancy also emerges as a noteworthy finding. In our study, the highest malaria prevalence was found in the second trimester (51.3%) and the lowest in the third trimester (15.5%). The relationship between the second trimester of pregnancy and malaria positivity was statistically significant (*p* = 0.011) (Tables 2 and 3). These findings are consistent with the studies of Odikamnoro et al. [36], Yakubu et al. [37], and Abdullahi et al. [38] support the higher malaria risk in the second trimester, while Melariri et al. [19] and Simon-Oke et al. [25] observed a higher risk in the first trimester. There are also studies reporting the highest prevalence in the third trimester [22, 39]. These discrepancies may stem from seasonal variation or differences in study population characteristics.

Although there was no statistically significant difference between blood groups and malaria prevalence in this study, it was shown that malaria positivity was much higher in pregnant women with O Rh+ blood group than in other blood groups (66.4%) (Table 2). While our result is similar to two studies in Gambia and Malawi [40, 41] that reported an association between blood group O and placental malaria, in another study [42], pregnant women with blood group O were more resistant to malaria infection than other blood groups.

Another important sociodemographic variable associated with malaria is the educational level of pregnant women. There are several studies showing that there is a relationship between the educational status of pregnant women and the prevalence of malaria. A study in northwestern Ethiopia reported that the susceptibility to malaria was increased in illiterate pregnant women compared to women with secondary and higher education [28]. Similar results were reported in studies conducted in Nigeria [43] and in Benishangul Gumuz in western Ethiopia [22]. In this study, when the prevalence of malaria was evaluated according to educational status; while the prevalence of malaria was similar in pregnant women with a college degree (31.5%) and those without education (31.1%), the lowest prevalence rate was found in pregnant women with a university degree (10.5%).

Numerous studies have demonstrated that the use of preventive measures significantly reduces malaria infection rates [22, 24]. In this study, the effect of using a method to prevent malaria on malaria positivity was found to be statistically significant (*p* < 0.001) (Tables 2 and 3). Pregnant women who used ITN and spray (10.1%) had lower malaria positivity during pregnancy compared to pregnant women who did not use ITN (79.8%). This result is consistent with studies showing that the use of ITN is highly effective in reducing malaria incidence in two different regions of eastern Nigeria, West Ethiopia, northwestern Uganda, northwestern Nigeria, and western Kenya [19, 22, 24, 44, 45]. However, according to studies in Burkina Faso [34] and the Jawi region in northwestern Ethiopia [46] the association between bed net use and malaria is not significant.

The high prevalence of malaria among symptomatic pregnant women in this study is consistent with the pathophysiology of *P. falciparum* infection. The blood stage of the parasite triggers the host's immune response, leading to the release of cytokines such as TNF-*α* and IL-1, which cause typical symptoms such as fever, chills, and headache [47]. In this study, the malaria positivity rate (71.8%) in symptomatic pregnant women participating in the study was significantly higher than in asymptomatic pregnant women (Table 2). Consistent with our study, in a study conducted in Northeastern Ethiopia [30], the malaria positivity rate in symptomatic pregnant women (48.4%) was higher than the malaria positivity rate in asymptomatic pregnant women (10.2%). However, in another study conducted in Northwestern Ethiopia [48], malaria positivity in asymptomatic pregnant women (4.8%) was higher than malaria positivity in symptomatic pregnant women (5.5%).

The overall prevalence of anemia among pregnant women participating in this study was found to be 67.6% (261/398). The World Bank research from 2016 on the “prevalence of anemia among pregnant women in Somalia” (46.8%) found that this prevalence was somewhat lower [49]. In another study, this rate was found to be 44.4% (170/383) [50]. In our study, 67.6% of pregnant women who tested positive for malaria were anemic similar to the study conducted in West Guji District of Ethiopia (66.4%) [51]. This rate was considerably higher, although not statistically significant (Table 2), but lower than a study in the Merti District of Ethiopia (92.3%) [52]. In malaria endemic areas, *Plasmodium* infection is one of the main causes of anemia. However, *Plasmodium* infection is not the only cause of anemia. Other causes such as malnutrition and helminthiasis may also cause anemia. However, studies have shown that *Plasmodium* infection in pregnant women can significantly worsen maternal anemia [51, 53]. In malaria endemic areas, *Plasmodium* infection is one of the main causes of anemia [54]. However, *Plasmodium* infection is not the only cause of anemia. Other causes, such as malnutrition, may also cause anemia [55]. These findings underscore the importance of integrating anemia prevention and management into national malaria control strategies, particularly in pregnancy. The high anemia prevalence observed aligns with WHO recommendations emphasizing routine screening and iron supplementation in antenatal care, especially in malaria-endemic regions. Incorporating these findings into regional programs can strengthen maternal health interventions and improve outcomes in vulnerable populations.

### 4.1. Limitations of Study

1. The study was conducted over a limited 3-month period (November–January), coinciding with peak malaria transmission season. This may have introduced seasonal bias, as data were not collected during periods of lower transmission.
2. Important factors such as the use of intermittent preventive treatment in pregnancy (IPTp) and frequency of antenatal care (ANC) visits were not assessed, although both are known to influence malaria risk and detection in pregnant women.
3. Due to limited laboratory resources, polymerase chain reaction (PCR)-based diagnostics were not used. As microscopy has lower sensitivity, particularly for low parasitemia or asymptomatic cases, some infections may have gone undetected, potentially underestimating malaria prevalence.

## 5. Conclusion

This study provides critical evidence on the substantial burden of malaria among pregnant women in Somalia, revealing a notably high prevalence rate of 59.8%, and underscores the urgent need for targeted interventions. The vast majority of cases (91.6%) were infected with the most severe clinical picture, *P. falciparum*. According to our findings, malaria positivity was most frequently seen in pregnant women in the second trimester (51.3%), while the lowest positivity rate was found in pregnant women in the third trimester (15.5%). In addition, the use of protective methods such as in IRS application and ITNs was significantly inversely associated with the risk of malaria infection. On the other hand, the rate of malaria infection in symptomatic pregnant women (71.8%) was found to be significantly higher than in asymptomatic pregnant women.

The findings highlight the importance of routine screening in pregnant women living in malaria-endemic areas. Routine malaria screening should be integrated into ANC, especially in the early stages of pregnancy. Furthermore, the dissemination of effective preventive strategies such as IRS and ITN is crucial to combating malaria. In this context, public health programs should prioritize educational interventions for pregnant women and ensure free and equal access to evidence-based malaria control tools. Future policies should also consider intermittent preventive treatment during pregnancy as part of comprehensive maternal health packages. These findings have important implications for national malaria control efforts, reinforcing WHO guidelines, informing ANC policies, guiding clinical practice, and identifying areas for future research to improve maternal and neonatal outcomes in malaria-endemic regions.

## Figures and Tables

**Table 1:** Sociodemographic and disease-related characteristics (*n* = 398).

**Variables**	**Categories**	**n**	**%**
Malaria	Positive	238	59.8
	*P. falciparum*	218	91.6
	*P. vivax*	20	8.4

Age groups	16–20	55	13.8
	21–30	242	60.8
	31–40	95	23.9
	41–50	6	1.5

Gravidity	Primigravidae	129	32.4
	Multigravidae	269	67.6

Gestational age	First trimester	130	32.7
	Second trimester	187	47.0
	Third trimester	81	20.4

Blood group	0 RH (−)	8	2.0
	0 RH (+)	250	62.8
	A RH (−)	4	1.0
	A RH (+)	96	24.1
	B RH (+)	33	8.3
	AB RH (+)	7	1.8

Marital status	Married	391	98.2
	Others (divorced/single)	7	1.8

Residence	Rural	16	4.0
	Urban	382	96.0

Educational status	No formal education	126	31.7
	Primary school	108	27.1
	Secondary school	127	31.9
	Higher education	37	9.3

Previous history of malaria	Yes	306	76.7
	No	92	23.1

Status of using a method to prevent malaria	Not used	267	67.1
	Indoor residual spray (IRS) utilization	58	14.6
	Insecticide-treated bed net (ITN) utilization	73	18.3

Symptom status	Asymptomatic	201	50.5
	Symptomatic	197	49.5

Clinical symptoms	Fever	60	30.4
	Headache	94	47.7
	Muscle joint pain	35	17.8
	Nausea, vomiting, diarrhea	8	4.1

Anemia status	Anemia	261	65.6
	Non-anemia	137	34.4

**Table 2:** Factors affecting malaria among pregnant women.

**Variables**	**Categories**	**Malaria**				**p**
		**Positive**		**Negative**		
		***n***	**%**	***n***	**%**	
Age groups	16–20	31	13.0%	24	15.0%	0.737
	21–30	150	63.0%	92	57.5%	
	31–40	54	22.7%	41	25.6%	
	41–50	3	1.3%	3	1.9%	

Gravidity	Primigravity	83	34.9%	46	28.7%	0.201
	Multigravity	155	65.1%	114	71.3%	

Gestational age	First trimester	79	33.2%	51	31.9%	**0.011**⁣^∗^
	Second trimester	122	51.3%	65	40.6%	
	Third trimester	37	15.5%	44	27.5%	

Blood group	0 RH (−)	3	1.3%	5	3.1%	0.409
	0 RH (+)	158	66.4%	92	57.5%	
	A RH (−)	2	0.8%	2	1.3%	
	A RH (+)	52	21.8%	44	27.5%	
	B RH (+)	20	8.4%	13	8.1%	
	AB RH (+)	3	1.3%	4	2.5%	

Marital status	Married	234	98.3%	157	98.1%	1.00
	Others (divorced/single)	4	1.7%	3	1.9%	

Residence	Rural	11	4.6%	5	3.1%	0.456
	Urban	227	95.4%	155	96.9%	

Educational status	No formal education	74	31.1%	52	32.5%	0.794
	Primary school	64	26.9%	44	27.5%	
	Secondary school	75	31.5%	52	32.5%	
	Higher education	25	10.5%	12	7.5%	

Previous history of malaria	Yes	179	75.2%	127	79.4%	0.334
	No	59	24.8%	33	20.6%	

Status of using a method to prevent malaria	Not used	190	79.8%	77	48.1%	**p** < 0.001^∗^
	IRS	24	10.1%	34	21.3%	
	ITN	24	10.1%	49	30.6%	

Symptom status	Asymptomatic	67	28.2%	134	83.8%	**p** < 0.001^∗^
	Symptomatic	171	71.8%	26	16.3%	

Anemia status	Anemia	161	67.6%	100	62.5%	0.289
	Nonanemia	77	32.4%	60	37.5%	

*Note:* Chi-square test and Fisher exact test: *a* = 0.05.

Abbreviations: IRS: indoor residual spray utilization; ITN: insecticide-treated bed net utilization.

⁣^∗^Distributional difference is statistically significant (bold).

**Table 3:** Logistic regression analysis.

**Variables**	**B**	**Wald**	**p**	**OR (95% CI)**
Age (reference)		1.998	0.573	
Age (21–30)	0.059	0.004	0.950	1.061 (0.167–6.723)
Age (31–40)	−0.371	0.175	0.676	0.690 (0.121–3.925)
Age (41–50)	−0.142	0.025	0.875	0.868 (0.147–5.104)
Gravidity	−0.258	0.836	0.360	0.772 (0.444–1.344)
Trimester (reference)		7.447	**0.024**⁣^∗^	
Trimester (second)	−0.647	4.052	**0.044**⁣^∗^	0.524 (0.279–0.983)
Trimester (third)	−0.817	7.361	**0.007**⁣^∗^	0.442 (0.245–0.797)
Blood group (reference)		5.006	0.415	
Blood group (0 Rh+)	0.396	0.122	0.727	1.485 (0.161–13.700)
Blood group (A Rh−)	−0.784	0.893	0.345	0.456 (0.090–2.321)
Blood group (A Rh+)	−0.561	0.168	0.682	0.571 (0.039–8.324)
Blood group (B Rh+)	−0.391	0.214	0.643	0.676 (0.129–3.545)
Blood group (AB Rh+)	−0.870	0.929	0.335	0.419 (0.071–2.457)
Place of residence	−0.330	0.298	0.585	0.719 (0.220–2.346)
Status of using a method to prevent malaria (reference)		40.654	**p** < 0.001^∗^	
Status of using a method to prevent malaria (IRS)	−1.652	31.397	**p** < 0.001^∗^	0.192 (0.108–0.342)
Status of using a method to prevent malaria (ITN)	−0.337	0.788	0.375	0.714 (0.340–1.502)
Anemia status	−0.430	3.196	0.074	0.651 (0.406–1.042)

*Note:* Age reference: 16–20 age group; pregnancy reference: primigravida; trimester reference: first trimester; blood group reference: O RH (−); reference for place of residence: rural; reference to the case of using a method to prevent malaria: no; anemia status reference: anemic; logistic regression analysis: *a* = 0.05; Nagelkerke *R*^2^:0.199.

Abbreviations: *B*, regression coefficient; IRS, indoor residual spray utilization; ITN, insecticide-treated bed net utilization; OR, odds ratio; Wald, test statistic value.

⁣^∗^The effect is statistically significant compared to the reference group (bold).

## Data Availability

The data used to support the findings of this study are included within the article. Further inquiries can be directed to the corresponding author.
